# Microvascular decompression for hemifacial spasm involving the vertebral artery: A modified effective technique using a gelatin sponge with a FuAiLe medical adhesive

**DOI:** 10.1111/cns.13662

**Published:** 2021-05-28

**Authors:** Fei Xue, Zhaoli Shen, Yuhai Wang, Sze Chai Kwok, Jia Yin

**Affiliations:** ^1^ Department of Neurosurgery Shanghai Tenth People's Hospital Tongji University Shanghai China; ^2^ Department of Neurosurgery Wuxi China; ^3^ Shanghai Key Laboratory of Brain Functional Genomics Key Laboratory of Brain Functional Genomics Ministry of Education School of Psychology and Cognitive Science East China Normal University Shanghai China; ^4^ Shanghai Key Laboratory of Magnetic Resonance East China Normal University Shanghai China; ^5^ NYU‐ECNU Institute of Brain and Cognitive Science at NYU Shanghai Shanghai China

**Keywords:** gelatin, microvascular, vertebral

## Abstract

Microvascular Decompression for Hemifacial Spasm Involving the Vertebral Artery (VA): A Modified Effective Technique Using a Gelatin Sponge with a FuAiLe Medical Adhesive. (a)The VA pushes the anterior inferior cerebellar artery (AICA) which compressed the root exit zone (REZ) of the facial nerve. (b) The VA was adhered to the petrous dura, and the AICA was decompressed from the REZ by a Teflon pad.

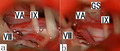

## CONFLICT OF INTEREST

None.

Hemifacial spasm (HFS) is a facial movement disorder characterized by involuntary, unilateral, and intermittent contractions of the facial muscles. These symptoms are embarrassing and uncomfortable, and they seriously affect quality of life. The patients also suffer from many social and psychological problems. Neurovascular conflict (NVC) has been considered the main cause of HFS in the root exit (or entry) zone (REZ) of the facial nerve in the cerebellopontine angle cistern.^1^, ^2^ Microvascular decompression (MVD) is widely accepted as an effective surgical method to treat HFS. Thousands of operations are carried out worldwide each year.^3^ In HFS, the most common offending vessels (OVs) in 75% of cases are the posterior inferior cerebellar artery (PICA) and anterior inferior cerebellar artery (AICA). For such patients, the MVD technique is used to achieve satisfactory results with Teflon taps. The REZ of the facial nerve is from the pontomedullary sulcus to the pontine surface and is close to the vertebral artery (VA). Therefore, a quarter of the OVs of HFS are VAs.^4^ If adding the numbers involving the VA (OVs are due to pushing by the VA or branching of the VA and only a transfer of the VA can achieve decompression), the rate of the VA as the OV of HFS is higher. When the VA is the OV, the diameter is larger; thus, the high blood flow in the above vessel also increases the pressure on the vessel wall. A curved OV experiences high tension due to the strong impact of blood flow. Additionally, the room for transfer is narrow. However, the Teflon decompression technique remains a challenge. Moreover, many previous studies have shown that MVD of HFS involving VA will exhibit higher rates of an incomplete cure and complications than those with the AICA or PICA.^5^

Many methods have been used to solve the above problem in recent years. However, these techniques are often dangerous, complex, time‐consuming, and increase the risk of surgical complications; therefore, they are not practical enough.^6^, ^7^, ^8^, ^9^, ^10^, ^11^ We introduce a simple and effective method using a gelatin sponge absorbed with a FuAiLe medical adhesive (FAL) to transfer the OV involving the VA.

This retrospective analysis included 87 patients with HFS who underwent their first surgical treatment consecutively between March 2015 and February 2019 at the author's department. Among them, 33 cases transferred the OVs with the FAL in MVD surgery when the OVs were involved with VAs. The other 54 cases were decompressed with the established Teflon tap technique. The general accident characteristics are listed in Table 1. Magnetic resonance imaging was performed preoperatively. Pure tone audiometry was carried out to identify any hearing impairments before and after surgery. All of the patients were correctly diagnosed, and serious primary diseases were excluded. All 87 patients had at least a 1‐ to 5‐year follow‐up.

**TABLE 1:** General accident characteristics, surgical complications, and operative outcomes of the two groups

	FAL (*n* = 33)	Teflon (*n* = 54)	*χ* ^2^ or *t*	*P*
M/F	14/19	29/25	1.04	0.31
Age	51.15 ± 12.59	53.41 ± 13.40	0.78	0.44
Duration	3.90 ± 2.38	4.81 ± 2.70	1.59	0.12
Follow‐up (year)	3.57 ± 1.50	3.09 ± 1.26	1.61	0.11
Cohen grade (3/4)	9/24	11/43	0.55	0.46
Hearing loss	4	7	0.01	0.91
Transient facial paralysis	6	5	1.47	0.22
Symptoms of Ⅸ–Ⅺ cranial nerves	3	6	0.09	0.76
Early remission	25	35		
Delayed remission	8	11		
Partial remission	0	6		
Ineffective	0	2		

**FIGURE 1:**
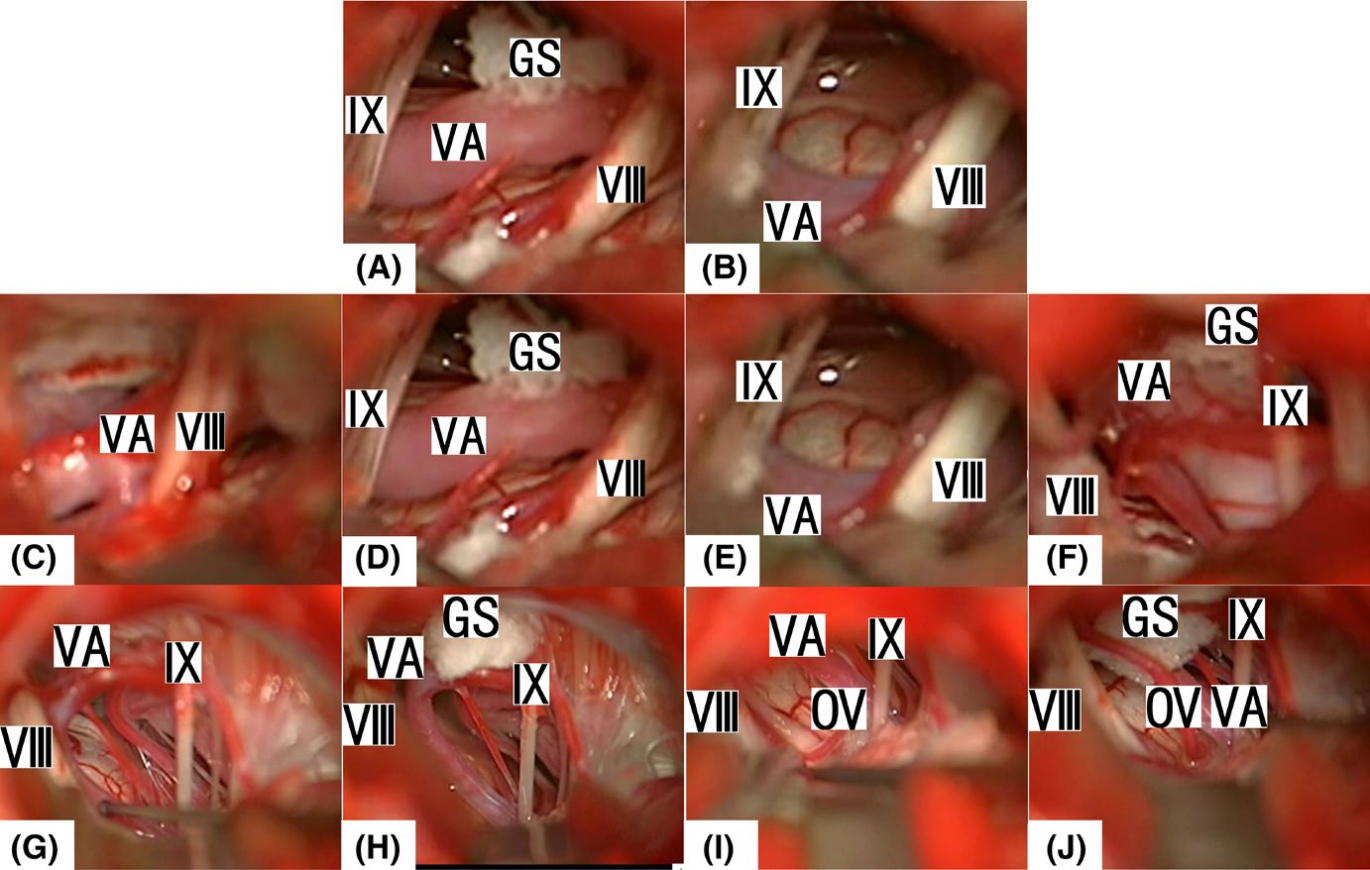
Intraoperative images of types I–IV. A, After opening the subtonsillar trans‐cerebellomedullary fissure, a VA compressed the REZ of a facial nerve. B, The VA as an OV was adhered to the petrous dura, and the REZ was decompressed. C, A branch of the VA compressed the REZ of a facial nerve. D, The VA and its OV branch were adhered to the petrous dura. A sufficient decompression of the REZ was achieved. E, The AICA was pushed by the VA, which compressed the REZ of the facial nerve. F, The VA was adhered to the petrous dura, and the AICA was decompressed from the REZ by a Teflon pad. G, An AV was found to compress the REZ. H, The AV was adhered to the petrous dura, and a branch of AICA was found to compress the REZ as the real OV. I, A Teflon pad was placed to lift the distal of the AV from the surface of brain stem. J, After the transposition of the real OV to the VA, sufficient decompression of the whole CN VII intracranial segment was achieved

A conventional retrosigmoid approach was used in the MVD for HFS with a patient in the lateral decubitus position. The inferior basal aspect of the cerebellum was retracted from caudal to rostral. The arachnoid membrane between the acoustic nerve and the Ⅸ, Ⅹ, and Ⅺ cranial nerves was dissected to expose the REZ of the facial nerve at the pons. The location of the OV was identified at the REZ. The Teflon group used a conventional Teflon pad to decompress between the brain stem and the OV. The FAL group had slightly different procedures according to the different situations involving OVs compressing the REZ, and they could be divided into 4 types: I. Direct compression by the VA; II. Compression by branches of the VA; III. The VA pushes a branch of the AICA or PICA which becomes an OV; IV. Adhesion the VA gives a space to decompress the small OV. The procedure includes the following steps. First, the OV was dissected and freed. The VA was pushed to the petrous dura to find a zone for adhesion, and then, the dura zone was electrocoagulated by bipolar coagulation to make it rough. Second, a gelatin sponge tap was prepared. It should be a dry, thin layer with dimensions of approximately 1 × 2 × 4 mm (the thickness can be determined according to the distance from the dura to the VA). FAL (Beijing FuAiLe Science and Technology Development Co., Ltd., Beijing, China) was applied to half of the tap. Third, after aspiration of the surrounding cerebrospinal fluid, the gelatin sponge tap (the half of FAL) was placed into the space between the dura and VA. Gun tweezers were used to clip the other half of the gelatin sponge tap without FAL. Fourth, the VA was pushed to the dura and held for several seconds to make sure that the VA firmly adhered to the dura. The last step was examining the ample space to decompress the REZ of the facial nerves from the OV (Figure 1).

Follow‐up information was obtained through phone interviews and a review of the medical records from clinic visits. The efficacy of MVD was categorized into 5 levels: early remission (complete and immediate disappearance of spasms), delayed remission (complete disappearance of spasms in 1 year), partial remission (slight spasms but better than that preoperatively after 1 year), failure (the same as that preoperatively after 1 year), and recurrence (spasms again after 1 year). A comprehension analysis of the surgical complications was performed postoperatively (in‐patient) and after 1 year (follow‐up visit). Permanent complications were compared, and transient complications were defined those as appearing in the patient postoperatively but that resolve after 1 year. The complications included facial paralysis, hearing loss (>20 dB), and symptoms from the Ⅸ–Ⅺ cranial nerves (hoarseness, dysphagia, and choking). Statistical analyses were performed using SPSS version 18.0. Continuous variables are presented as the mean ±standard deviation, and categorical variables are presented as frequency (%). Age, sex, Cohen grade, duration, follow‐up, surgical outcomes, and complications were compared across groups using chi‐square tests, unpaired *t* tests, and Wilcoxon rank sum tests. A *P*‐value less than 0.05 was considered to indicate significant differences between groups.

All the patients had an uneventful postoperative recovery and were discharged for home on the 7th postoperative day in good condition. The surgical outcomes and complications of the two groups are shown Table 1. There was no recurrence in either group. None of the patients experienced convulsion, poor wound healing, infection, CSF leakage, meningitis, or hydrocephalus as an adverse reaction. Other reported complications were as follows.

Table 1. General accident characteristics, surgical complications, and operative outcomes of the two groups.

No significant differences were observed in terms of gender, age, Cohen grade, and duration or in the postoperative follow‐up or complications between the two groups. The Wilcoxon rank sum test was used to compare the operative outcomes of the two groups, and the significance was calculated as *Z* = 2.53, *p* = 0.01.

Since Jenetta in 1966, it has been generally accepted that HFS is caused by a compression of the facial nerve at the REZ; thus, neurosurgeons generally perform neurovascular decompression at the REZ. In recent decades, MVD has been widely applied in clinical practice owing to its safety and efficacy.^1^, ^12^

The key for MVD is achieving a sufficient decompression. By exploring the REZ area of the cranial nerve, the OV is found, transferred, and fixed to prevent it from nearing the REZ. The most commonly used method to achieve decompression is the use of some material (usually a Teflon pad) as a fulcrum and raising the OV. The method of decompression for HFS is different from that for trigeminal neuralgia (TGN). MVD for TGN can be achieved only by separating the nerves from the OV by the Teflon pad. However, MVD for HFS requires the use of the Teflon pad as a bridge pier and the brain stem surface as the ground; in this way, the OV is lifted from the brain stem through the Teflon pier and becomes the bridge deck. The bridge needs to maintain a certain height so that the REZ of the facial nerve can flow smoothly, like water in a river. When appropriate decompression is provided, long‐term spasm relief is achieved.^13^, ^14^ If the bridge pier is not high enough, collapses or is improperly distanced between the two piers, it may collapse, similar to a bridge, and then recompress the REZ of the facial nerve, which is the main cause of failure and recurrence.

When the OV is a smaller artery such as the AICA or PICA, the bridge using the above method is easy to build, and the surgical outcome is satisfactory. However, in clinical practice, because of the anatomical location of the VA, which is close to the REZ of the facial nerve, the VA is one of the most common OVs of HFS. In some instances, the VA pushes one of the small arteries (PICA or AICA or one of their branches) near the REZ, especially when it becomes tortuous and atherosclerotic with age.^15^ In this case, the usual method using material such as a Teflon pad as the bridge pier does not work well, although it appears satisfactory intraoperatively. The reason why is because the bridge piers made of the Teflon pad may move or collapse with tension, which can be caused by the pulse of the VA, or by the cerebellum resetting postoperatively. The Teflon pad, VA or branches of the VA will then again exert pressure on the REZ of the facial nerve. The outcome is an ineffective operation. Thus, a safe and reliable method to transfer the VA and firmly fix the OV to avoid its regression on the REZ has been actively explored by surgeons in recent years. There are several types of methods:

1. Fixation with an instrument, such as the use of fenestrated aneurysm clips to fix the VA to a distant place
2. The use of complicated approaches to transfer the VA at the proximal segment (a suboccipital far‐lateral approach)
3. Wedge technique for transposition of the VA
4. Chemical glue
5. Biomedical glue‐coated Teflon sling transposition

These techniques are either difficult to conduct (it is difficult to put an aneurysm clip and other cords to tie the VA in such a small space) or result in a large amount of trauma, prolonging the surgical and anesthesia times. In addition, the use of liquid glue is not easy to control, and chemical irritation from the glue may cause aseptic meningitis, as it can flow throughout the surgical area. Moreover, if the operative device touches the glue and then touches the vessels and nerves, they can bond together and be difficult to separate.^13^ As a result, these techniques are not satisfactory, and most have been reported in only a small number of cases. Furthermore, the rate of surgical complications also increases. In 2017, Zhang reported fairly good results, with 174 cases using bioprotein glue.^16^ Recently, because the adhesive technique with glue is simpler and safer, many doctors have considered adhesion to be the better choice to transfer and fix the VA and as an alternative method to the bridge technique. In addition, although bioprotein glue has better tissue compatibility and is safer than liquid chemical glue, the adhesion of FAL (a chemical glue) is stronger and adheres firmly. This is the key for decompression.

Avoiding the shortcomings of FAL as a chemical liquid and giving full consideration to the advantages of strong adhesion is similar to changing rocket fuel from a liquid to a solid to improve safety. We managed to achieve this through the use of a piece of gelatin sponge as a carrier that absorbed FAL. After transferring the VA from the REZ of the facial nerve, a FAL‐absorbed gelatin sponge was placed between the dura and the VA, and then, pressure was administered for several seconds. The VA stuck tightly to the dura, and the decompression purpose was successful. This method avoids the uncontrollable flow of liquid glue, and the technique is simple, efficient, and safe. Compared with the control group using conventional Teflon decompression during the same period, the FAL technique shows a higher efficiency and fewer complications, making it an ideal technique to prevent the VA from approaching the facial nerve REZ.

Technical notes:

1. The gelatin sponge should be cut to a suitable size according to the adhesion space; furthermore, the area that is clipped by forceps must contain no FAL. Otherwise, it is difficult to withdraw the forceps.
2. If some surgical instruments are stuck to the FAL, then there can be no touching of the brain tissue, nerves, or blood vessels before being wiped and cleaned.
3. Sometimes, the operator must add in a drop of FAL to enhance the fixation. A plastic dropper must be used instead of a syringe because a syringe cannot precisely control the volume. In most cases, one drop is enough. An excessive amount of FAL can flow to the subarachnoid space, and the chemical stimulation will cause meningitis and cranial nerve damage.
4. The OV and their artery perforators must be carefully protected from damage during transferring. All arteries must not be bent at such an angle that it affects blood flow after being adhered. If the distance between the OV and the dura is too great, then the thickness of the gelatin sponge should be increased, which will help protect and avoid artery perforators.
5. The first application is best. It is difficult to adjust the sponge after the FAL is adhered.

This method can also be extended to other situations: 1. There were many artery perforators at the location of the Teflon pad, or there were other factors that led to difficulty in placing the pad. 2. The transfer of the VA to the petrous dura could produce a space for the real OV to decompress the REZ. Small OVs can also be pasted to the VA indirectly. This method is more suitable for types 3 and 4.

This technique is relatively simple and has many advantages, but it is not a panacea. If decompression after adhesion is still unsatisfactory, a Teflon pad can be inserted to achieve satisfactory decompression. In summary, the key techniques and principles for the MVD are that the OV is transferred and fixed firmly away from the REZ.

When the OVs of hemifacial spasms involve the VA, a decompression technique with a gelatin sponge and a FAL medical adhesive is effective, simple, and safe.
